# PCR-Induced Transitions Are the Major Source of Error in Cleaned Ultra-Deep Pyrosequencing Data

**DOI:** 10.1371/journal.pone.0070388

**Published:** 2013-07-23

**Authors:** Johanna Brodin, Mattias Mild, Charlotte Hedskog, Ellen Sherwood, Thomas Leitner, Björn Andersson, Jan Albert

**Affiliations:** 1 Department of Microbiology, Tumor and Cell Biology, Karolinska Institutet, Stockholm, Sweden; 2 Department of Virology, Swedish Institute for Infectious Disease Control, Stockholm, Sweden; 3 Science for Life Laboratory Stockholm, Solna, Sweden; 4 Theoretical Biology and Biophysics, Los Alamos National Laboratory, Los Alamos, New Mexico, United States of America; 5 Department of Cell and Molecular Biology, Karolinska Institutet, Stockholm, Sweden; Duke-National University of Singapore Graduate Medical School, Singapore

## Abstract

**Background:**

Ultra-deep pyrosequencing (UDPS) is used to identify rare sequence variants. The sequence depth is influenced by several factors including the error frequency of PCR and UDPS. This study investigated the characteristics and source of errors in raw and cleaned UDPS data.

**Results:**

UDPS of a 167-nucleotide fragment of the HIV-1 SG3Δenv plasmid was performed on the Roche/454 platform. The plasmid was diluted to one copy, PCR amplified and subjected to bidirectional UDPS on three occasions. The dataset consisted of 47,693 UDPS reads. Raw UDPS data had an average error frequency of 0.30% per nucleotide site. Most errors were insertions and deletions in homopolymeric regions. We used a cleaning strategy that removed almost all indel errors, but had little effect on substitution errors, which reduced the error frequency to 0.056% per nucleotide. In cleaned data the error frequency was similar in homopolymeric and non-homopolymeric regions, but varied considerably across sites. These site-specific error frequencies were moderately, but still significantly, correlated between runs (r = 0.15–0.65) and between forward and reverse sequencing directions within runs (r = 0.33–0.65). Furthermore, transition errors were 48-times more common than transversion errors (0.052% vs. 0.001%; p<0.0001). Collectively the results indicate that a considerable proportion of the sequencing errors that remained after data cleaning were generated during the PCR that preceded UDPS.

**Conclusions:**

A majority of the sequencing errors that remained after data cleaning were introduced by PCR prior to sequencing, which means that they will be independent of platform used for next-generation sequencing. The transition vs. transversion error bias in cleaned UDPS data will influence the detection limits of rare mutations and sequence variants.

## Background

Ultra-deep pyrosequencing (UDPS), which is one of the applications of next-generation sequencing (NGS), offers new possibilities to detect minority sequence variants [1], [2], [3], [4]. UDPS involves sequencing of very large numbers of single DNA template molecules that usually have been generated by a preceding PCR. UDPS is therefore also known as amplicon sequencing or targeted resequencing. Until the introduction of next-generation sequencing, Sanger sequencing was the dominating sequencing technology. Sanger sequencing has also been applied to collections of non-identical DNA templates, so called population sequencing, for instance for routine genotypic HIV resistance testing [5]. However, population Sanger sequencing can only detect minority variants that represent more than 10–20% of a heterogeneous sequence population (e.g. a HIV-1 quasispecies) [6], [7]. This restricted sequencing depth sometimes limits research and clinical utility. Thus, minority HIV resistance mutations, below the detection limit of population Sanger sequencing, have been shown to be of clinical relevance [8], [9], [10], [11], [12]. The importance of sequencing depth has also been shown in studies of rare cancer cells in biopsies [13].

The resolution of UDPS is primarily determined by the number of input DNA templates and the error frequency of the method. In this context it is a draw-back that UDPS has higher error frequency than Sanger sequencing (approximately 0.5% vs. 0.1% errors per nucleotide site) [14], which means that it may be difficult to distinguish rare, but genuine, sequence variants from sequencing artefacts. The type of sequencing errors also differs between UDPS and Sanger sequencing. Homopolymeric regions, i.e. runs of the same nucleotide, pose a particular problem during pyrosequencing because there is no terminating signal to prevent multiple consecutive incorporations at a given cycle. Therefore the length of homopolymers is inferred from differences in light intensity, which become increasingly smaller as a function of homopolymer length [14], [15]. UDPS errors due insertions and deletions (indels) are therefore over-represented in homopolymeric regions [16]. The indel errors are primarily generated during the emission, detection and interpretation of the chemi-luminescent light signal that is generated during pyrosequencing [14]. However, UDPS errors can also be introduced by other mechanisms, such as nucleotide misincorporations and indels during PCR or uneven nucleotide-flow over the Picotiter plate. The 454-sequencing software removes reads with some types of errors, e.g. reads originating from two or more DNA templates, but both indel errors and substitution errors may be present in the UDPS data that is output from the instrument, herein referred to as “raw” UDPS data. Therefore, researchers have used different bioinformatic approaches to identify as well as remove or correct these sequencing artefacts [17], [18], [19], [20], [21]. Several of these data cleaning procedures have reduced UDPS error frequencies down to 0.05%, but there is still incomplete knowledge about the character of the errors that remain after data cleaning as well as in which steps of the sequencing procedure they are introduced.

Here we present a comprehensive investigation of the types and frequencies of errors that occurred when the UDPS was used to repeatedly sequence an HIV-1 molecular clone. Data cleaning reduced the average error frequency from 0.30% to 0.056%. Most errors that remained after data cleaning were transitions that primarily were introduced PCR rather than during the actual UDPS. The difference in frequency of transition vs. transversion errors will lead to site-specific differences in the detection limits of minority mutations.

## Results

### UDPS Data and Definitions of Sequencing Errors

In this study we have investigated the types and frequencies of errors that occur during repeated UDPS of an HIV-1 clone (SG3Δenv). The investigation consisted of two parts. First we characterized sequencing errors in the UDPS data that is output from the instrument, herein referred to as “raw” UDPS data. Based on these analyses and previous publications [16], [19], [22], we developed a set of scripts (Text S1) that filtered reads that were likely to contain sequencing errors. Second, we characterized the sequencing errors that remained after we had applied our UDPS data cleaning strategy.

The target amplicon consisted of a 167-base pair fragment of the HIV-1 *pol* gene corresponding to amino acids 170–224 of the reverse transcriptase. The SG3Δenv clone was subjected to three separate, bidirectional UDPS runs and we obtained a total of 47,693 UDPS reads (*Table 1*). As shown in *Table 1*, we divided these reads into six datasets corresponding to the reads in forward or reverse sequencing direction from each of the three UDPS run.

**Table 1:** Total number of reads and mean error frequency percent (%) per nucleotide as well as the number of unique sequence variants in raw and cleaned UDPS data from forward and reverse reads from three UDPS runs.

		Raw data		Cleaned data	
Run	Sequencing direction	No. of reads/mean % errorfrequency per nucleotide	No. of uniquevariants	No. of reads/mean % errorfrequency per nucleotide	No. of uniquevariants
1	Forward	10,121/0.20	633	8,756/0.063	204
	Reverse	7,378/0.19	480	6,205/0.058	146
2	Forward	12,092/0.23	682	9,537/0.058	206
	Reverse	10,482/0.61	527	1,462/0.077	91
3	Forward	2,570/0.21	271	2,187/0.041	85
	Reverse	5,050/0.14	354	4,583/0.08	124
Total	Both	47,693/0.30	2,044	32,730/0.056	315

**Figure 1:**
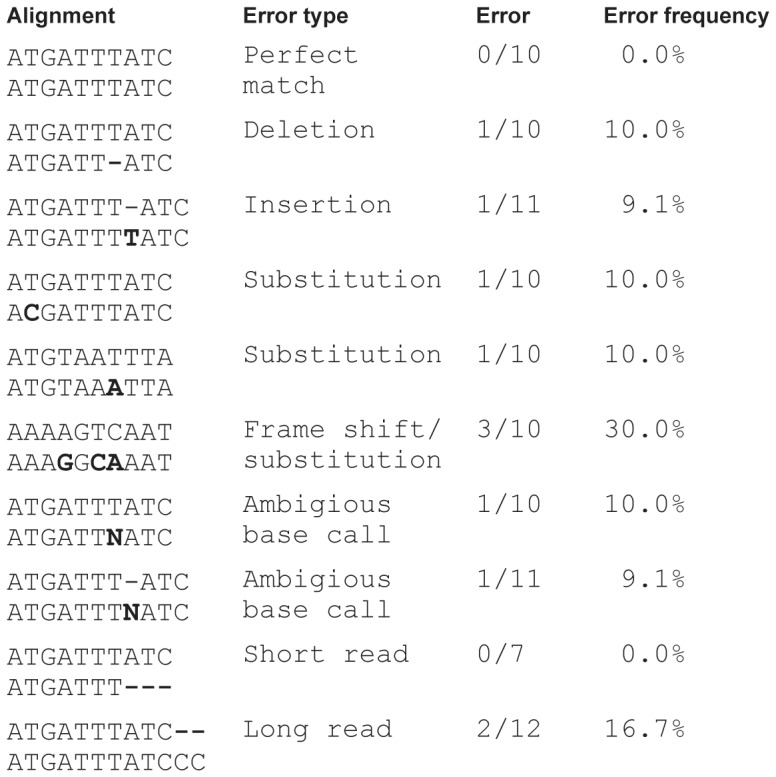
Examples of how different types of UDPS error were defined.

An UDPS read was defined as having one or more sequencing errors if the sequence of the read did not exactly match the published sequence of the SG3Δenv clone, which was identical to a *de novo* Sanger population sequence of the target region of the clone. All sequences were aligned in a multiple alignment created by Genome Amplicon Variant Analyzer (454 Life Sciences, Branford, CT). Sequencing errors were categorized as substitutions, deletions or insertions as illustrated in *Figure 1*. We separately investigated sequencing errors in homopolymeric and non-homopolymeric regions of the amplicon.

### Errors in Raw UDPS Data

The average error frequency in the raw UDPS data from the three runs was 0.30% per nucleotide. Deletions were the most frequent error type (56%) and had an average per nucleotide frequency of 0.16%. Insertions and substitutions constituted 24% and 20% of the errors, respectively, and had an average per nucleotide frequency of 0.069% and 0.057%. We also separately studied the error frequencies in homopolymeric and non-homopolymeric regions. The error frequency was higher in homopolymeric regions (0.59% per nucleotide) than in non-homopolymeric regions (0.12% per nucleotide), but this difference was not statistically significant for any of the six datasets (p = 0.14–0.90, Mann-Whitney U-test). Sequencing errors were distributed unevenly also within homopolymeric and non-homopolymeric regions. The site-specific frequencies of the positions containing deletion errors ranged from 0.0021% to 20.39% in homopolymeric regions and from 0.0021% to 0.086% in non-homopolymeric regions, whereas the frequencies of insertion errors ranged from 0.0021% to 1.25% in homopolymeric regions and from 0.0021% to 1.36% in non-homopolymeric regions. Finally, the site-specific frequencies of substitution errors ranged from 0.0021% to 0.17% in homopolymeric regions and from 0.0063% to 1.17% in non-homopolymeric regions. The type of substitution errors were also distributed unevenly. *Table 2* shows that transition errors and especially G→A and T→C were more common than transversion errors in raw UDPS data. *Table 2* also shows that there were considerable differences in substitution error frequencies across sites. For instance, for G→A substitutions the site-specific error frequency ranged from 0% (i.e. no substitution observed) to 0.21%.

**Table 2:** Frequency of specific nucleotide substitution errors in raw UDPS data.

	To base			
From base	A	T	G	C
A	–	0.00 (0.00–0.01)	0.06 (0.00–0.20)	0.00 (0.00–0.02)
T	0.00 (0.00–0.01)	–	0.00 (0.00–0.03)	0.06 (0.00–0.19)
G	0.02 (0.00*–0.21)	0.00 (0.00–0.01)	–	0.00 (0.00–0.01)
C	0.00 (0.00–0.01)	0.02 (0.00–0.18)	0.00 (0.00–0.01)	–

Results were combined from the three UDPS runs and are displayed as median and range percent (%) error per nucleotide.

0.00* denotes an error frequency of = 0.00021%. 0.00 denotes that the substitution error was not observed.

The length distribution of the total dataset of 47,693 UDPS reads is shown in *Figure S1*. A total of 33,092 (69%) of the reads had the expected length of 167 bases, whereas 11,562 (24%) reads were shorter than expected and 3,039 (6%) reads were longer than expected. A majority (83%) of the short reads occurred among the reverse reads from run 2, in which 8,525 of 10,482 (81%) of the reads lacked an adenosine (A) in a homopolymeric stretch of six A's from position 145 to 151 of the amplicon. This sequencing error was probably introduced during pyrosequencing since only 118 of 12,092 (0.96%) of the forward reads of the same PCR product displayed this deletion. Sequencing errors were strongly associated with read length (Table S1). Most (29,852 of 33,264; 90%) reads with the expected length (167 bases) were correct, which resulted in an average error frequency of 0.07% errors per nucleotide. In contrast all 2,867 reads that were longer than expected had at least one insertion error and had an average error frequency of 1.13% per nucleotide. Finally, 99% of all reads that were shorter than 167 nucleotides (11,436 of 11,562 reads) had at least one deletion (often the missing A mentioned above) and their average error frequency was 0.71% per nucleotide.

### Over-representation of PCR-induced Transition Errors in Cleaned UDPS Data

**Figure 2:**
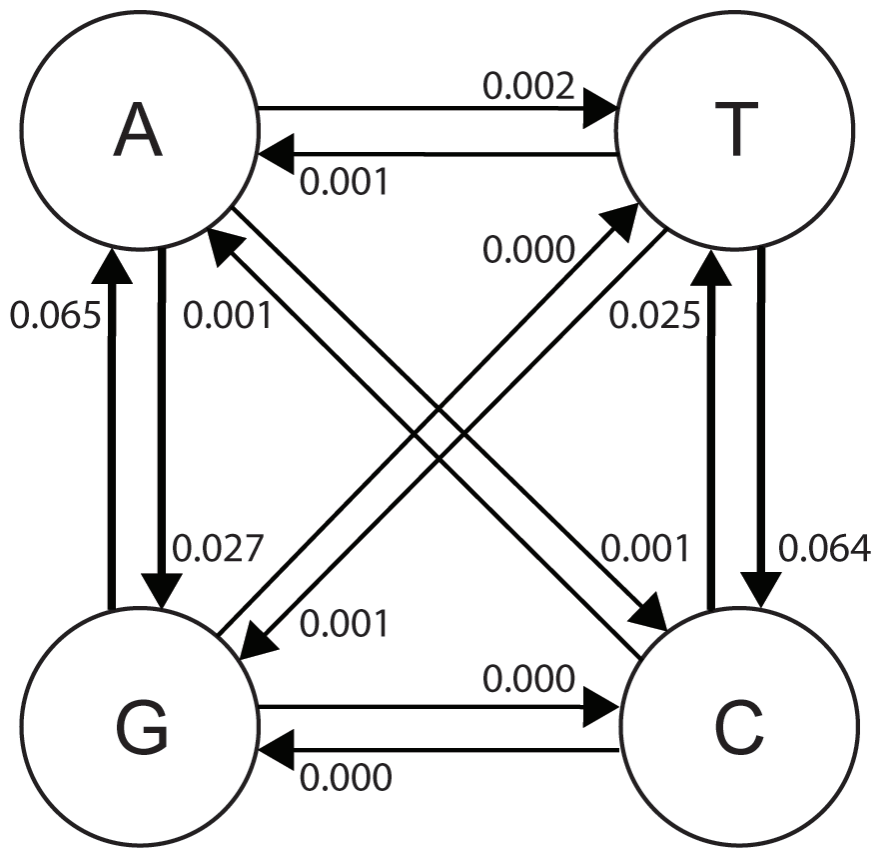
The average frequency of different substitution errors in percent (%) in cleaned UDPS data from three sequencing runs. Thick arrows indicate transitions and thin arrows indicate transversions.

We used an in-house data cleaning strategy with scripts that removed 14,963 of 47,693 (31%) of reads based on presence of indels (except indels involving entire codons), unresolved bases (N’s) and stop codons (see Text S1). This removed all, except two, of the reads with indel errors. These two reads had three A’s inserted in a homopolymeric region of five A’s and where retained because our cleaning strategy did not filter reads with insertions of entire codons. In contrast, the frequency of substitution errors was largely unaffected by the data cleaning. Consequently, the average error frequency per nucleotide for the six datasets after data cleaning was 0.056% (range 0.038–0.077%) (*Figure S2, Table S2*), which was similar to the frequency of substitution errors in raw data (0.057%). In all three sequencing runs and both sequencing directions, the error frequencies in cleaned data were similar in homopolymeric and non-homopolymeric regions (average 0.054% vs. 0.057% per nucleotide; p = 0.34–0.81, Mann-Whitney U-test). Transition errors, especially G→A and T→C transitions, were over-represented in the cleaned UDPS data compared to transversion errors (*Figure 2*). The average frequency of transition errors was 0.052% per nucleotide and the average frequency of transversion errors was 0.0001%, a 48-fold difference (p<0.001, Fisher exact test). As discussed further below, this transition/transversion bias indicated that most substitution errors were generated during the PCR that preceded UDPS.

**Figure 3:**
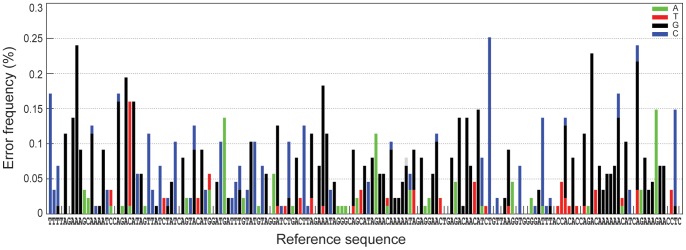
Site-specific error frequencies in percent (%) in cleaned UDPS data obtained in the forward sequencing direction of run 1. All sequencing errors were substitutions since all deletions and insertions were removed by the data cleaning procedure. The bars are color-coded according to the type of substitution error. Homopolymeric regions are shaded.

Next, we investigated the error frequency across nucleotide sites in the cleaned UDPS data. As shown in *Figure 3* *,* the site-specific errors varied considerably as exemplified by the forward reads in run 1 where the site-specific errors varied from a minimum of 0% per site to a maximum of 0.25% per site. Similar results were obtained in the forward reads from the two other runs as well as the reverse reads from all three runs. To further characterize these errors we studied the correlation of site-specific error frequencies between the forward runs, the reverse runs, and the forward and reverse reads from the same run. There were moderate, but significant, correlations of site-specific error frequencies in forward as well as reverse reads in all three 454 runs (Spearman R = 0.31–0.65; p<0.001) (*Table S3)*. The same was true when the forward and reverse sequencing direction was compared within runs (Spearman R = 0.33–0.60; p<0.001). In contrast, we found no evidence for a general increase in error frequency across the 167-base pair amplicon (data not shown). The correlation between errors in forward and reverse reads suggests that systematic errors were introduced during PCR, which agrees well with the strong bias towards transition errors.

**Figure 4:**
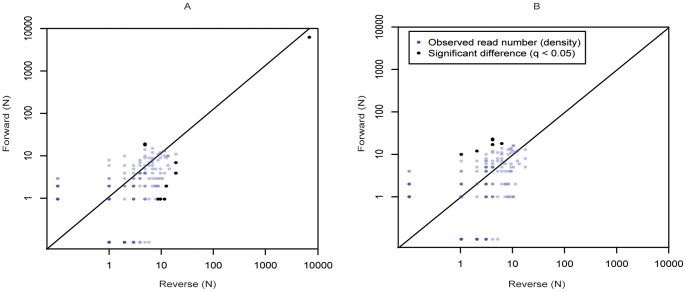
PCR/UDPS error ratio in our cleaned data. This figure shows a comparison of the counts of reverse to forward variants of run 1. A) Our filtered UDPS data. B) Same data, normalized by the main variant forward and reverse counts.

Because we cannot expect every PCR product to be available or sequenced in the 454-system, the counts of each sequence variant detected will be stochastic regardless of whether it is a genuine variant or a variant generated due to sequencing errors. Naturally, this stochastic effect will be more severe at low counts, such as the low-frequency sequencing errors that remained after data cleaning. Hence, we next asked if the forward and reverse read counts for specific variants were within an expected Poisson margin. The forward vs. reverse read counts that are significantly different are most likely not due to PCR errors, but rather UDPS induced. The ratio of significantly and non-significantly correlated errors in forward and reverse reads of the same run thus gives us a measure of the proportion of PCR vs. UDPS errors. We used a q-value ≤0.05 to account for the false discovery rate that arises from the large number of p-values analyzed. The PCR/UDPS error ratio was 217/11 = 19.7, 157/64 = 2.4, and 146/2 = 73 in runs 1, 2, and 3, respectively, after our cleaning procedure (*Figure 4* *)*. Hence, these analyses also indicated that the vast majority of the errors that remained after data cleaning occurred during the pre-UDPS PCR amplification. Interestingly, while our cleaning procedure reduced the overall differences between forward and reverse counts (from 940 to 228 in run 1, from 1088 to 221 in run 2, and from 526 to 148 in run 3), it cleaned up PCR errors proportionally more in runs 1 and 2 (ratio changed from 26.6 to 19.7 and 10.1 to 2.45) and vice versa in run 3 (ratio changed from 51.6 to 73). If only PCR errors occurred, then a normalization using the main variant of the forward and reverse counts should remove all differences (within a Poisson expectation). However, if differences remain, they are likely UDPS errors. Indeed, when we normalize each run the remaining significant differences between forward and reverse counts diminish from 11 to 6, 64 to 15, and 2 to 0, respectively, further indicating that most errors that remain in our data after cleaning originate in the pre-UDPS PCR amplification.

Finally, we calculated the expected contribution of errors due to PCR based on the number of PCR cycles and error rate of the PCR enzyme (i.e. FastStart High Fidelity PCR System with a reported error rate of approximately 4.0 * 10^−6^). The probability for a single position to be incorrect after 60 PCR cycles is 1-(1–4.0 * 10^−6^)^60^ = 0.024%. This agrees well with the results above that showed that PCR contributes to a substantial proportion of errors that remain in our cleaned UDPS data.

## Discussion

In this study we have investigated the type, frequency and source of sequencing errors in raw and cleaned UDPS data from a molecular clone corresponding to a fragment of the HIV-1 *pol* gene. As reported by others, UDPS-induced indels in homopolymeric regions was the dominating error type in raw UDPS data [16], [18]. In contrast, a substantial proportion of the errors that remained after data cleaning were substitutions errors introduced in the PCR that preceded UDPS. These substitution errors significantly more often were transitions than transversions.

In line with our findings, Shao *et al*. recently reported that substitution errors in raw UDPS data were mainly introduced during PCR [23]. Here we extend this finding by showing that PCR contributed to a substantial proportion of substitution errors also in cleaned UDPS data. Thus, our data cleaning strategy, which effectively removed indel errors, had little effect on PCR-induced substitution errors. This problem may be relevant also to other cleaning pipelines. However, some programs, like PyroNoise and AmpliconNoise, attempt to remove both sequencing and PCR errors [20], but it unclear how well they perform on highly diverse viral data. Importantly, our finding that PCR contributes to a substantial proportion of sequencing errors in cleaned UDPS data is relevant to studies that utilize other NGS platforms. Thus, the Illumina NGS platform, which is gaining popularity in HIV research because it has lower error rates and higher throughput than the 454 platform, also requires PCR amplification of HIV templates prior to NGS. Our findings indicate that HIV sequences generated on the Illumina platform can be expected to have an error rate of approximately 0.05% per nucleotide unless additional measures are taken to reduce PCR-induced errors. Examples of such measures could include reduction of the number of PCR cycles and use of DNA polymerases with even higher fidelity than the polymerase mix that we used. It is well-known that PCR error frequency increases with increasing number of PCR cycles [24], [25], but to our knowledge the effect of reduced number of PCR cycles on NGS error frequencies has not been thoroughly investigated. However, Zagordi *et al.* and Shao *et al.* reported significantly reduced UDPS error frequencies in clones that were not PCR amplified prior to UDPS [17] [23]. A problem with using polymerases with very high fidelity is that they tend to have low processivity, which may reduce PCR success and thereby sequence depth. In addition, high fidelity enzymes have been reported to increase PCR recombination frequency [23]. The Primer ID technology, which involves tagging and resequencing of individual template molecules [26], represents an interesting new approach to further reduce NGS error frequency.

We found that transition errors were 48-times more common than transversion errors in cleaned UDPS data. This overrepresentation of transition errors in UDPS has been observed in two other studies [23], [27]. The transition/transversion bias is typical polymerase misincorporations where the T•G mispairings are over-represented [24], [28], [29], [30], [31]. It is unlikely that errors introduced during the pyrosequencing steps should have this substitution bias despite the fact that a polymerase is used as part of the enzyme mixture in the emulsion PCR. This is because each DNA temple is bound to a single microbead and by emulsion PCR used to generate millions of DNA templates that are subjected to “consensus” sequencing. Thus, the overrepresentation of transversion errors, as well as our other results, shows that a substantial proportion of the UDPS errors that remained after data cleaning were introduced during PCR. The strong transition/transversion bias in cleaned UDPS data has implications for detection of minority mutations, for instance minority HIV-1 resistance mutations. Thus, our findings indicate that different cut-offs are needed for detection of mutations involving transitions compared to transversions.

In summary, we have investigated the frequency, type and source of errors that occurred during UDPS of a fragment of the HIV-1 pol gene. A substantial proportion of the errors that remained after data cleaning were introduced in the pre-UDPS PCR amplification and they significantly more often were transitions than transversions, which affects the limits of detection of minority mutations. Our findings are of relevance to other NGS applications and platforms, because PCR errors will be introduced will be independent of NGS platform, as long as sequencing is preceded by PCR.

## Materials and Methods

### UDPS Data

The data for this work were generated as part of a published study by Hedskog *et al*. [4]. The data consisted of 47,693 UDPS reads of the SG3Δenv plasmid that were generated in three separate runs on the Genome Sequencer FLX (454 Life Sciences, Branford, CT) (*Table 1*). The plasmid is available at the NIH AIDS Research and Reference reagent Program under catalogue no. 11051 and the sequence of the parent plasmid pSG3.1 is available in Genbank under accession no. L02317. The amplicon contained 167 nucleotides from the HIV-1 pol gene corresponding to the last nucleotide of amino acid 169, amino acids 170–224, and the first nucleotide from amino acid 225 as well as the sample tags and the 454-specific adaptors A and B. Briefly and as described previously, single molecules of the SG3Δenv plasmid were obtained by limiting dilution and amplified using nested PCR (30+30 cycles) using a polymerase mix with high fidelity (Roche FastStart High Fidelity System). The amplicon was subjected to bidirectional UDPS. The entire procedure from sample preparation to UDPS was repeated on three separate occasions. The sequence of the targeted regions of the plasmid clone was determined by Sanger sequencing and was identical to the published sequence.

Sequence analyses were performed on the total dataset of 47,693 UDPS reads as well as separately for forward and reverse reads from each of the three UDPS runs. Furthermore, for each UDPS read we created two concatenated sub-sequences that combined all homopolymeric and non-homopolymeric regions, respectively. There exists no formal definition of a homopolymeric region, but here we defined a homopolymeric region as a stretch of at least three identical nucleotides as well as one preceding and one following nucleotide. *Figure 3* shows the homopolymeric and non-homopolymeric regions in the amplicon.

### Programming

The scripts for data management, data cleaning and sequence analyses were written in Perl. We used the collection of biological applications from BioPerl [32]. We also used interfaces from EMBOSS package [33] to get methods for sequences alignment. Gnuplot was used to develop a visualization tool for an easy overview of the data. The data cleaning scripts were inspired by Tsibris *et al*. [*19*] *,* who kindly made their code available prior to publication and the results from analyzing our own raw data. Our scripts filtered reads with: 1) less than 80% similarity to a user-defined reference sequence, 2) ambiguous nucleotide calls, 3) indels, and 4) stop codons (Text S1). The scripts are available at http://ki.se/ki/jsp/polopoly.jsp?d=23336&a=34965&l=en.

### Definition of Sequencing Errors and Calculation of Error Frequencies

Any difference from the Sanger sequence of the SG3Δenv plasmid in the UDPS analysis was defined as a sequencing error. The sequencing errors were classified as deletions, insertions or substitutions as described in the Results section and in *Figure 1*. The average UDPS error frequency per nucleotide was estimated from the three sets of UDPS data. The Needleman-Wunsch algorithm with gap opening score 10 and gap extend score 0.5 was used to construct pairwise alignments between the Sanger sequence of the SG3Δenv plasmid as a references sequence and UDPS reads from the plasmid. The identity score, the number of correctly aligned bases divided by the total number of bases, from the pairwise comparisons were added together and divided by the number of sequences and the 95% confidence interval was calculated. For reads that were shorter than 167 bases we only compared positions that were actually sequenced (see Figure 1).

Additionally, site-specific error frequencies were estimated individually for all nucleotide positions. Again the Sanger sequence of the SG3Δenv plasmid was used as a reference sequence and compared to the UDPS reads from all three runs. The error in each position was divided by the number of reads in the same position to get the error frequency and the 95% confidence interval. The script has to rely on a previous alignment or sequences that do not contain indels.

### Analysis of Type of Sequencing Errors

To identify and study the types of UDPS sequencing errors that were encountered we used the same approach as for the calculations of site specific error frequency. Thus, an alignment constructed by the GS Amplicon Variant Analyzer Software was used. A deletion error was defined as a gap in the alignment of an UDPS read relative to the Sanger reference sequence. An insertion error was defined as a gap in the reference sequence that was absent in the UDPS read. A substitution error was defined as a nucleotide difference between the UDPS read relative to the references sequence. The frequencies of different substitution (G→A, G→C, etc) were calculated by dividing the number of substitutions between specific nucleotides and the number of possible substitutions of those nucleotides.

### Statistical Analyses

Statistical analyses were performed in Statistica version 10 and R version 2.10.1. Comparisons of error frequencies in homopolymeric and non-homopolymeric regions were done using the Mann-Whitney U-test. Correlations of site-specific error frequencies between runs as well as between forward and reverse reads in the same run were done using the Spearman rank correlation test (*Table S3*). The use of this test, which requires independent observations, was justified because we did not observe any significant correlations in error frequencies of adjacent nucleotides (range 1–5) or excess error frequencies in transitions between homopolymeric and non-homopolymeric regions (data not shown). To obtain p-values for the Spearman correlations we created a null distribution of 100,000 Spearman R-values by randomizing (100,000 times) the site-specific error frequencies of one dataset while keeping those of the other dataset constant (e.g. errors from the forward reads of run 2). P-values for observed Spearman R-values were calculated using a z-test. Finally, the frequency of transition vs. transversion errors was compared using the Fisher exact test. The observed number of transitions and transversions were compared with their expected numbers, where we expected that there should be twice as many transversions if there was no transition vs. transversion substitution bias. Because substitution errors that occur during PCR may be amplified in subsequent PCR cycles, we conservatively counted each observed substitution only once.

## Supporting Information

Figure S1 UDPS read length distribution. The expected read length of 167 bases is shown in black.
(TIF)

Figure S2 Flow chart showing steps of the UDPS data error cleaning procedure. Shown is also the number of reads that were filtered and that remained, respectively, as well as their average error frequency in percent (%) for the complete sequence, the homopolymeric regions and the non-homopolymeric regions.
(TIF)

Table S1 Influence of read length on sequencing errors in three bidirectional UDPS runs of a 167-base pair long fragment of the SG3Δenv HIV-1 plasmid.
(DOCX)

Table S2 Number of remaining reads and mean error frequency percent (%) per nucleotide when the filtering steps of the cleaning strategy were consecutively applied to raw data from three runs of bidirectional UDPS of the SGΔenv HIV-1 plasmid.
(DOCX)

Table S3 Table showing results from z-test used to generate p-values for Spearman rank correlations between site-specific error frequencies in different UDPS runs and sequencing directions. Null distributions of Spearman R values were generated as described in Materials and methods.
(DOCX)

Text S1 Description of the UDPS data error cleaning procedure and the impact of each filtering step.
(DOCX)
