# Supplementary figures and images for: PCR-Induced Transitions Are the Major Source of Error in Cleaned Ultra-Deep Pyrosequencing Data
Source: PLoS One. 2013 Jul 23;8(7):e70388. doi: 10.1371/journal.pone.0070388 (PMC3720931; doi:10.1371/journal.pone.0070388)

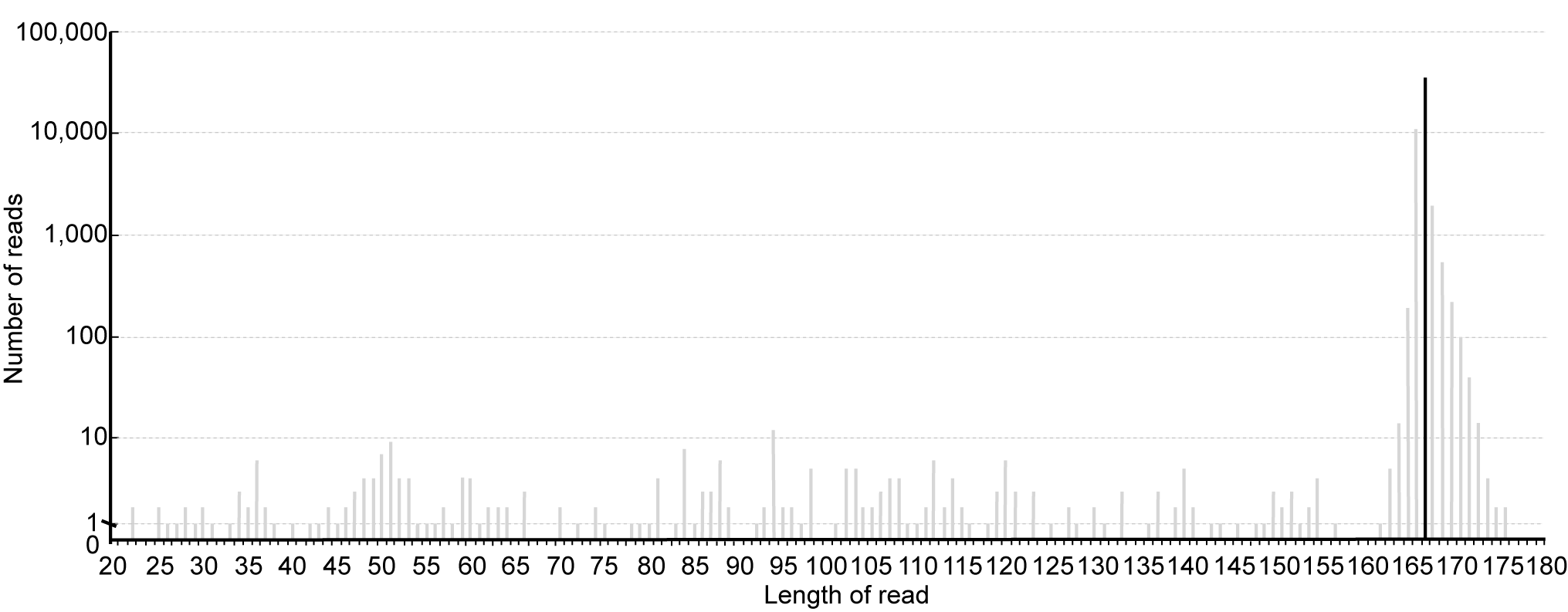

Supplement: Figure S1 — UDPS read length distribution. The expected read length of 167 bases is shown in black. (TIF) [file pone.0070388.s001.tif]

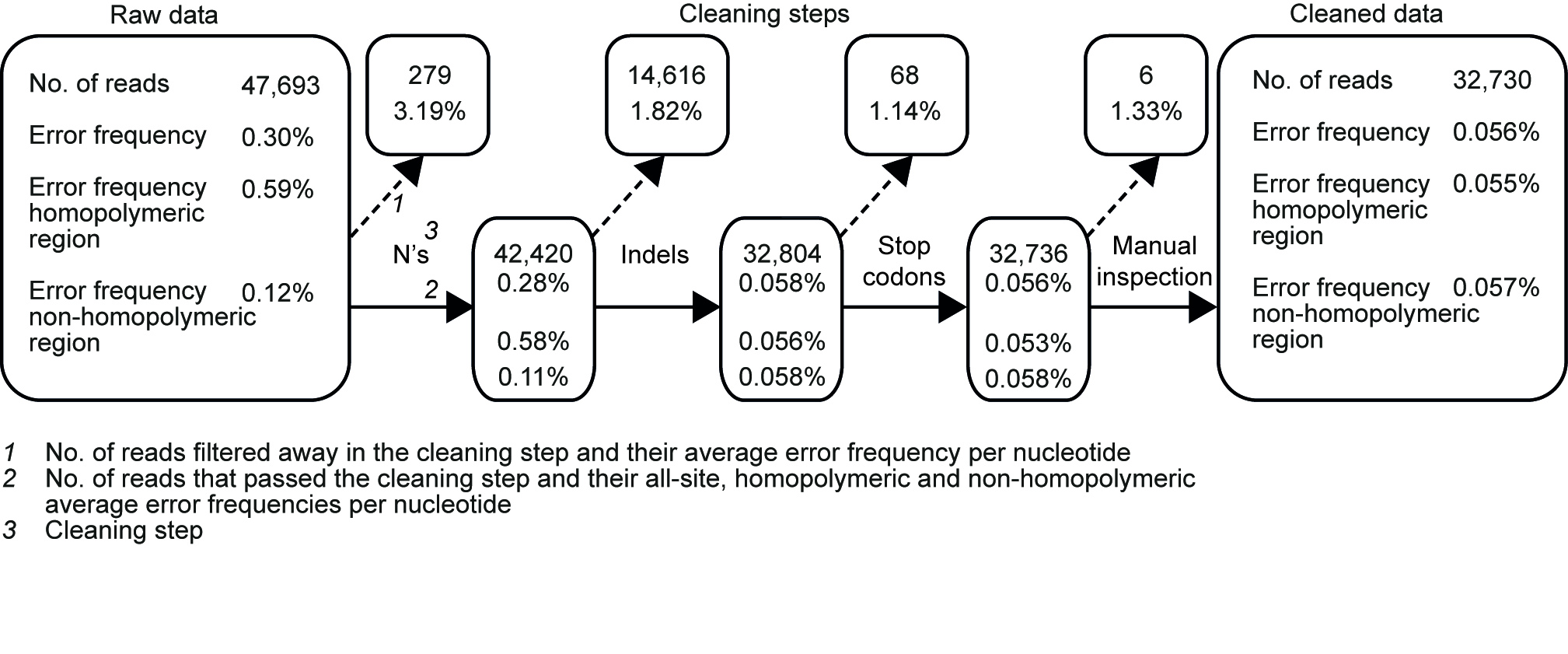

Supplement: Figure S2 — Flow chart showing steps of the UDPS data error cleaning procedure. Shown is also the number of reads that were filtered and that remained, respectively, as well as their average error frequency in percent (%) for the complete sequence, the homopolymeric regions and the non-homopolymeric regions. (TIF) [file pone.0070388.s002.tif]
