# Supplementary material for: PCR-Induced Transitions Are the Major Source of Error in Cleaned Ultra-Deep Pyrosequencing Data
Source: PLoS One. 2013 Jul 23;8(7):e70388. doi: 10.1371/journal.pone.0070388 (PMC3720931; doi:10.1371/journal.pone.0070388)
Supplement: Table S1 — Influence of read length on sequencing errors in three bidirectional UDPS runs of a 167-base pair long fragment of the SG3Δenv HIV-1 plasmid. (DOCX) [file pone.0070388.s003.docx]

**Table S1.** Influence of read length on sequencing errors in three bidirectional UDPS runs of a 167-base pair long fragment of the SG3Δenv HIV-1 plasmid.

|  | |  | |  | No. of reads with errors / total no. of reads | | |
| --- | --- | --- | --- | --- | --- | --- | --- |
| Run | Sequencing direction | | Total no. of reads | | Read length  < 167 bases | Read length = 167 bases | Read length > 167 bases |
| 1 | | Forward | | 10,121 | 436 / 461 | 914 / 8,843 | 817 / 817 |
|  |  | Reverse | | 7,378 | 446 / 460 | 644 / 6,267 | 651 / 651 |
| 2 | | Forward | | 12,092 | 1,653 / 1,683 | 970 / 9,627 | 782 / 782 |
|  |  | Reverse | | 10,482 | 8,643 / 8,648 | 349 / 1,637 | 197 / 197 |
| 3 | | Forward | | 2,570 | 104 / 131 | 186 / 2,232 | 207 / 207 |
|  |  | Reverse | | 5,050 | 154 / 179 | 349 / 4,658 | 213 / 213 |
| Total | | Both | | 47,693 | 11,436 / 11,562 | 3,412 / 33,264 | 2,867 / 2,867 |
