# Supplementary material for: PCR-Induced Transitions Are the Major Source of Error in Cleaned Ultra-Deep Pyrosequencing Data
Source: PLoS One. 2013 Jul 23;8(7):e70388. doi: 10.1371/journal.pone.0070388 (PMC3720931; doi:10.1371/journal.pone.0070388)
Supplement: Table S2 — Number of remaining reads and mean error frequency percent (%) per nucleotide when the filtering steps of the cleaning strategy were consecutively applied to raw data from three runs of bidirectional UDPS of the SGΔenv HIV-1 plasmid. (DOCX) [file pone.0070388.s004.docx]

### Table S2. Number of remaining reads and mean error frequency percent (%) per nucleotide when the filtering steps of the cleaning strategy were consecutively applied to raw data from three runs of bidirectional UDPS of the SGΔenv HIV-1 plasmid.

|  |  |  | Filtering step | | | | | |
| --- | --- | --- | --- | --- | --- | --- | --- | --- |
|  |  |  | (no. of remaining reads / mean % error frequency per nucleotide) | | | | | |
| Run | Sequencing direction | Raw UDPS data | 80% similarity | Ambiguous bases (N’s) | Indels | Stop codons | Manual removal of indels |  |
| 1 | Forward | 10,121/ 0.20 | 10,121/ 0.20 | 10,054/ 0.19 | 8,782/ 0.064 | 8,758/ 0.063 | 8,756/ 0.063 |  |
|  | Reverse | 7,378/ 0.19 | 7,378/ 0.19 | 7,320/ 0.19 | 6,219/ 0.060 | 6,208/ 0.059 | 6,205/ 0.058 |  |
| 2 | Forward | 12,092/ 0.23 | 12,092/ 0.23 | 12,085/ 0.23 | 9,550/ 0.059 | 9,538/ 0.058 | 9,537/ 0.058 |  |
|  | Reverse | 10,482/ 0.61 | 10,482/ 0.61 | 10,472/ 0.61 | 1,465/ 0.078 | 1,462/ 0.077 | 1,462/ 0.077 |  |
| 3 | Forward | 2,570/ 0.21 | 2,570/ 0.21 | 2,511/ 0.18 | 2,190/ 0.042 | 2,187/ 0.041 | 2,187/ 0.041 |  |
|  | Reverse | 5,050/ 0.14 | 5,050/ 0.14 | 4,972/ 0.12 | 4,598/ 0.040 | 4,594/ 0.038 | 4,583/ 0.08 |  |
| Total | Both | 47,693/ 0.30 | 47,693/ 0.30 | 47,414/ 0.28 | 32,804/ 0.058 | 32,737/ 0.056 | 32,730/ 0.056 |  |
