# Supplementary material for: PCR-Induced Transitions Are the Major Source of Error in Cleaned Ultra-Deep Pyrosequencing Data
Source: PLoS One. 2013 Jul 23;8(7):e70388. doi: 10.1371/journal.pone.0070388 (PMC3720931; doi:10.1371/journal.pone.0070388)
Supplement: Table S3 — Table showing results from z-test used to generate p-values for Spearman rank correlations between site-specific error frequencies in different UDPS runs and sequencing directions. Null distributions of Spearman R values were generated as described in Materials and methods. (DOCX) [file pone.0070388.s005.docx]

| **Table S3.** Spearman R values for correlations between forward and reverse sequence reads from the three UDPS runs (top row) and p-values generated by z-tests as described in Materials and Methods. | | | | | | | | | |
| --- | --- | --- | --- | --- | --- | --- | --- | --- | --- |
| UDPS data 1 | |  | UDPS data 2 | | | | | | |
|  |  |  | Forward sequences | | |  | Reverse sequences | | |
|  |  |  | Run 1 | Run 2 | Run 3 |  | Run 1 | Run 2 | Run 3 |
| Forward  sequences | Run 1 |  | - | 0.65 <0.00001 | 0.31  0.00003 |  | 0.60 <0.00001 | 0.45 <0.00001 | 0.47 <0.00001 |
|  | Run 2 |  |  | - | 0.34 <0.00001 |  | 0.65 <0.00001 | 0.40 <0.00001 | 0.56 <0.00001 |
|  | Run 3 |  |  |  | - |  | 0.36 <0.00001 | 0.15  0.0244 | 0.33 <0.00001 |
|  |  |  |  |  |  |  |  |  |  |
| Reverse  sequences | Run 1 |  |  |  |  |  | - | 0.44 <0.00001 | 0.52 <0.00001 |
|  | Run 2 |  |  |  |  |  |  | - | 0.33 <0.00001 |
|  | Run 3 |  |  |  |  |  |  |  | - |
