# Supplementary material for: PCR-Induced Transitions Are the Major Source of Error in Cleaned Ultra-Deep Pyrosequencing Data
Source: PLoS One. 2013 Jul 23;8(7):e70388. doi: 10.1371/journal.pone.0070388 (PMC3720931; doi:10.1371/journal.pone.0070388)
Supplement: Text S1 — Description of the UDPS data error cleaning procedure and the impact of each filtering step. (DOCX) [file pone.0070388.s006.docx]

### UDPS data filtering procedure

To decrease the UDPS error frequency we designed a set of scripts to filter UDPS data from reads that were likely to contain sequencing errors (*Figure S1*). The strategy was designed based on sequencing errors in raw UDPS data as well as previous publications [[16](#_ENREF_16), [19](#_ENREF_19), [22](#_ENREF_20)]. The data filtering strategy detects variation relative to a user-defined reference sequence, here a Sanger population sequence of the SG3Δenv plasmid. Each filtering step divided the sequence reads into two files; one file with reads that passed the filtering and another file with reads that were removed by the filtering because they had characteristics associated with an increased probability of containing sequencing errors. The scripts were designed for data cleaning of UDPS reads from coding parts of the HIV-1 genome and the cleaning strategy may not be directly applicable to all types of UDPS data. The steps in this data filtering procedure and their ability to reduce the UDPS error frequency are described below, in *Figure S1 and* *Table S2*. All steps are optional and some of the filtering parameters can be modified by the user. We applied the data filtering in the following order:

*Identification of unique UDPS reads*

Prior to filtering, we reduced the number of reads by identifying unique reads and enumerated their abundance in a new FastA file. Forward and reverse reads were handled separately. *Table 1* shows that this step reduced the total number of reads from 47,693 to 2,044, which considerably improved the overview of the data as well as simplified and speeded up all subsequent filtering steps and analyses.

### *Removal of low similarity reads*

The first filtering step removes reads with low similarity to a reference sequence, i.e. non-HIV sequences or HIV sequences of very low quality (*Figure S2*). We used the Needleman-Wunsch algorithm to construct pairwise alignments between a user-defined reference sequence, in this case the Sanger sequence of the SG3Δenv plasmid, and the unique UDPS reads to obtain the similarity score. If the alignment identity score is below a user-defined threshold the read is removed. Here we used a threshold of 80% identity. In our particular datasets the script did not identify any low similarity reads.

### *Removal of reads with ambiguous base calls “N’s”*

The 454-software uses the character “N” to describe an ambiguous base call. Huse et al showed that reads from the Genome Sequencer 20 (454 Life Sciences, Branford, CT) instrument containing N’s have a higher error frequency than reads without ambiguous base calls [[16](#_ENREF_16)]. This was observed also in our data that were generated using the GS-FLX instrument. Thus, 279 reads with N’s had an average error frequency of 3.19% per nucleotide, whereas reads without N’s had an error frequency of 0.28% per nucleotide. N’s occurred both in homopolymeric and non-homopolymeric regions (129 times and 155 times, respectively). We filtered reads containing N’s, which reduced the UDPS error frequency per nucleotide by 0.02%.

### *Removal of reads with out-of-frame indels*

UDPS errors frequently involve indels, especially in homopolymeric regions [15]. Therefore, we identified reads with out-of-frame indels and longer (≥6 nucleotides) frame-shifted regions. This step retained reads with indels involving entire codons as well as reads with short frame-shifted regions (<6 nucleotides), which may represent functional HIV-1 variants. The latter reads were flagged to allow visual inspection. As shown in *Figure S1*, this filtering step had a pronounced effect on the overall UDPS error frequency; we identified a total of 14,616 reads that were removed because of out-of-frame indels. This reduced the average error frequency almost 5-fold from 0.28% per nucleotide to 0.058% per nucleotide. As expected, the effect was most pronounced in homopolymeric regions, but it was also substantial in non-homopolymeric regions. After applying this filtering step the error frequencies did not differ between homopolymeric (0.056%) and non-homopolymeric (0.058%) regions (p=0.34-0.99, Mann-Whitney U-test). In addition, we manually removed six reads (representing four unique variants) with short frame-shifted regions that also had experienced substitution errors (see *Figure 1*) that were not identified by the script.

*Removal of reads with stop codons*

UDPS data from coding regions that contain stop codons are likely to represent sequencing errors, or are otherwise evolutionary dead-ends. In our UDPS data from the SG3Δenv plasmid, stop codons by definition were UDPS errors. After translation we found 68 reads with stop codons that had not already been removed by previous filtering steps. This step reduced the error frequency from 0.058% to 0.056% per nucleotide. We would not apply this filter if we would be interested in studying stop codons in UDPS data from clinical patient samples, nor if we studied non-coding regions.
